# Embolic occlusion of internal carotid artery in conscious rats: Immediate effects of cerebral ischemia

**DOI:** 10.14814/phy2.15613

**Published:** 2023-02-17

**Authors:** Thomas J. K. Toung, Noah Mehr, Marek Mirski, Raymond C. Koehler

**Affiliations:** ^1^ Department of Anesthesiology and Critical Care Medicine Johns Hopkins University Baltimore Maryland USA; ^2^ Present address: Department of Pathology University of Chicago, School of Medicine Chicago Illinois USA

**Keywords:** anesthesia, blood pressure, embolism, focal cerebral ischemia, rat, stroke

## Abstract

In most preclinical models of focal ischemic stroke, vascular occlusion is performed under general anesthesia. However, anesthetic agents exert confounding effects on mean arterial blood pressure (MABP), cerebrovascular tone, oxygen demand, and neurotransmitter receptor transduction. Moreover, the majority of studies do not use a blood clot, which more fully models embolic stroke. Here, we developed a blood clot injection model to produce large cerebral artery ischemia in unanesthetized rats. Under isoflurane anesthesia, an indwelling catheter was implanted in the internal carotid artery via a common carotid arteriotomy and preloaded with a 0.38‐mm‐diameter clot of 1.5, 3, or 6 cm length. After discontinuing anesthesia, the rat was returned to a home cage where it regained normal mobility, grooming, eating activity, and a stable recovery of MABP. One hour later, the clot was injected over a 10‐s period and the rats were observed for 24 h. Clot injection produced a brief period of irritability, then 15–20 min of complete inactivity, followed by lethargic activity at 20–40 min, ipsilateral deviation of the head and neck at 1–2 h, and limb weakness and circling at 2–4 h. Neurologic deficits, elevated MABP, infarct volume, and increased hemisphere water content varied directly with clot size. Mortality after 6‐cm clot injection (53%) was greater than that after 1.5‐cm (10%) or 3‐cm (20%) injection. Combined non‐survivor groups had the greatest MABP, infarct volume, and water content. Among all groups, the pressor response correlated with infarct volume. The coefficient of variation of infarct volume with the 3‐cm clot was less than that in published studies with the filament or standard clot models, and therefore may provide stronger statistical power for stroke translational studies. The more severe outcomes from the 6‐cm clot model may be useful for the study of malignant stroke.

## Graphical abstract

In a refinement of the embolic clot stroke model, injection of different length blood clots into the internal carotid artery of unanesthetized rats produced graded increases in arterial blood pressure, brain water content, neurological deficits, and infarct volume. The increase in arterial pressure correlated with the eventual volume of infarcted brain tissue.

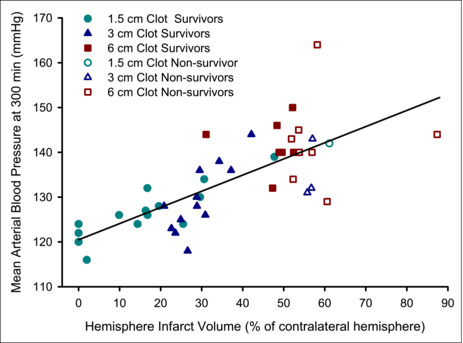

## 1 INTRODUCTION

To better understand the pathophysiologic mechanisms of focal cerebral ischemic stroke and to develop therapeutic targets for stroke, a variety of preclinical experimental models have been developed over the past few decades. Currently, the main ischemic stroke models used in rodents are the intraluminal filament model, the embolic clot model, the endothelin‐induced vasoconstriction model, and the distal middle cerebral artery (MCA) cauterization model (Fluri et al., 2015; Li & Zhang, 2021). The MCA cauterization model requires a craniotomy and results in permanent ischemia with no reperfusion. The intracranial injection of endothelin produces transient vasoconstriction with some inherent variability in the severity and duration of ischemia and also requires a craniotomy. The intraluminal filament model, in which a filament with an enlarged tip is inserted through the internal carotid artery (ICA) to wedge at the MCA orifice, has become the most commonly used model because it avoids the craniotomy and permits reperfusion at a well‐defined time. However, it does not mimic the interaction of an embolic clot with the endothelium and platelets (Zhang et al., 2001). Thus, large cerebral artery embolization with a blood clot has theoretical advantages for most closely mimicking clinical stroke.

To meet the need for a preclinical embolic clot model, Zhang, Chopp, et al. (1997) developed a model in which a preformed blood clot of fixed size was injected into the ICA of anesthetized rats to occlude the MCA (MCAo). This model has been useful for evaluating thrombolytic therapy (Zhang et al., 1998, 2004, 2009). However, this model has not become as a widely adopted as the intraluminal filament model, in part because of the considerable variability in the resulting infarct volume. In addition to the variability in collateral blood flow in both the filament and clot models, inconsistency in the clot model deployment among laboratories is partially attributable to the unpredictability in the quality of the clot (red or white, with or without thrombin), the size of embolus (25–55 mm in length), the use of a clot that is smaller in diameter than the vessel intended for occlusion, and the use of clot delivery catheter that is inconsistent in size and cross‐sectional shape.

Another important factor is the use of general anesthesia during the induction of ischemia. Because of effects of anesthetics on mean arterial blood pressure (MABP), cerebrovascular tone, cerebral oxygen consumption, and glutamatergic and GABAergic signaling, anesthetic agents may affect a variety of pathophysiological and biochemical determinants differently than those occurring in patients in the early stage of stroke. Acute hypertension is common in patients after acute ischemic stroke (AlSibai & Qureshi, 2016; Qureshi et al., 2007); those patients presenting with low blood pressure because of contracted blood volume can have worse neurologic scores (Bahouth et al., 2022), and raising blood pressure can reduce the perfusion/diffusion mismatch seen on MRI (Hillis et al., 2003). In preclinical studies, arterial blood pressure is often not monitored, especially after the animal emerges from anesthesia. There are a few studies in Wistar rats and spontaneously hypertensive rats undergoing the filament model of MCAo that reveal a rapid hypertensive response as the animals emerge from anesthesia, with hypertension persisting for 2 days (Thakkar et al., 2019, 2020). In the clot embolization model in Wistar rats, we previously showed that prolonged inhalation anesthesia beyond 30 min from the onset of clot embolization delayed the increase in MABP and was associated with greater cerebral edema and infarct volume (Chuang et al., 2018). Thus, anesthesia not only limits the observations of the early effects of ischemia on physiological and sensorimotor functions, but can alter the outcome in the embolic clot model.

In order to overcome these limitations associated with the current embolic MCAo model, and also to better mimic the pathogenesis of a large cerebral artery thromboembolic stroke in humans, we developed a new method of acutely occluding the internal carotid artery (ICA) by injecting a large diameter blood clot into the ICA through an indwelling catheter in the common carotid artery (CCA) of the conscious, normal functioning rat under normal systemic hemodynamic parameters. While validating the model's pathologic sequelae, the immediate effect on behavior and the arterial blood pressure response were also evaluated after injection of different size clots.

## 2 MATERIALS AND METHODS

The study was conducted in accordance with the National Institutes of Health guidance for the care and use of animals in research and the protocol was approved by the Animal Care and Use Committee of the Johns Hopkins University.

### 2.1 Preparation of blood clot

We used the method as described by Zhang, Chopp, et al. (1997) but with modifications. The femoral artery blood from a donor rat was withdrawn into a 40 cm long PE‐90 tube (internal diameter, ID, 0.86 mm), and retained in the tube for 3 h to clot at a temperature of 30–32°C. Subsequently the clot was stored for 22 h at 4°C. The PE‐90 tube, containing the clot, was then cut to a length of 1.5, 3, or 6 cm, after which the clot was flushed out into a petri dish containing normal saline to separate the clot from serum. The clot was then aspirated into a PE‐20 tube (ID, 0.38 mm) and flushed out into a saline‐filled petri dish. The latter step was repeated several times until a solid single, 1.5, 3, or 6 cm length clot was obtained. The clot was finally aspirated into a saline‐filled indwelling catheter (PE‐50, ID, 0.58 mm) with its tip marked in centimeters for 7 cm.

### 2.2 Surgical preparation

Anesthesia was induced with 4% isoflurane in adult, male Wistar rats (400–450 g; 3–4‐months old), and was thereafter maintained with 1%–2% isoflurane for femoral artery catheterization and placement of an indwelling catheter. The right femoral artery was cannulated with PE 50 tubing and exteriorized through a swivel device sutured to the upper back, between the two scapulae, for continuous monitoring of systemic MABP. The rat was then placed into a left lateral position in a modified stereotactic frame. Through a right lateral neck skin incision, the CCA was carefully dissected and externalized. The proximal CCA was lifted and gently retracted for optimal exposure of the external and internal carotid artery (ECA‐ICA) bifurcation. The occipital artery was carefully isolated, cauterized and severed. The pterygopalantine artery was identified and ligated with a 5‐0 silk suture at its origin at the ICA. The ECA and ICA were carefully separated, and ECA was ligated with 5‐0 silk. A 4‐0 silk suture was looped around ICA proximal to the origin of pterygopalantine artery for later retraction to prevent back bleeding from a CCA arteriotomy during insertion of indwelling catheter. A pre‐tie 5‐0 stay suture was placed around the proximal ICA (for later fixation of indwelling catheter). The proximal CCA was ligated with 4‐0 silk suture and the suture was kept for later fixation of indwelling catheter to CCA. A 4‐0 pre‐tie silk was placed around the distal CCA (for later closure of arteriotomy and fixation of indwelling catheter). A 16‐G 1–1/2 inch needle was inserted from the sternal head of sternocleidomastoid muscle (close to where the CCA emerged from the chest cavity), tunneled subcutaneously toward the right shoulder and exited by stabbing through the skin just cephalad to the right scapula. An indwelling PE‐50 catheter, with its tip marked in centimeters for 7 cm and loaded with a pre formed arterial blood clot, was introduced in a retrograde fashion from the tip of the 16G needle, and placed with the tip of the catheter next to the CCA. The catheter was inserted into the CCA via a small arteriotomy, advanced 2 mm into the ICA, and affixed with the previously placed 5‐0 silk suture. The catheter was also affixed to the CCA at the arteriotomy site, and further at the proximal CCA. The catheter was finally secured with a 4‐0 Teflon suture at the skin free of tension. The neck wound was closed. All surgical wounds were infiltrated with 0.5% bupivacaine for postoperative comfort. The rat was placed in a home cage allowing to recover from anesthesia.

### 2.3 Production of cerebral ischemia

**FIGURE 1:**
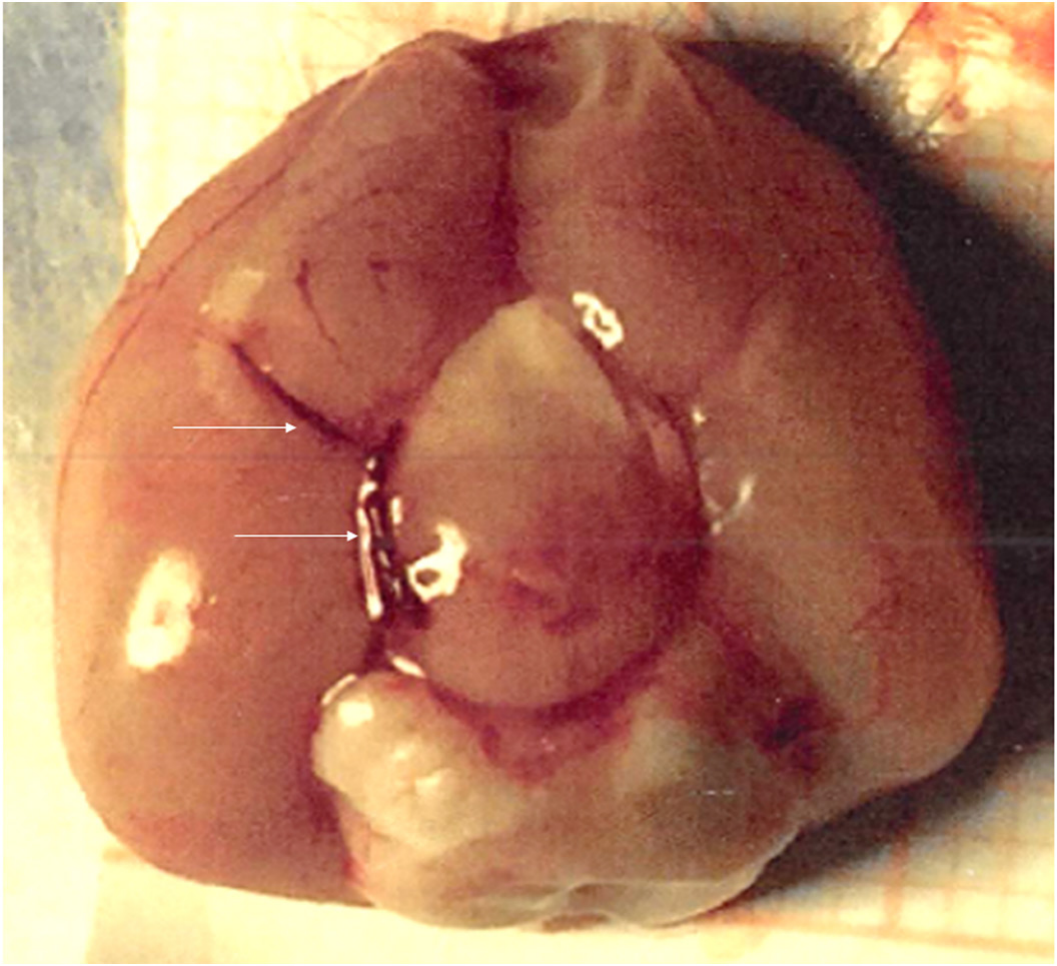
Photograph of a ventral surface of rat brain, showing the blood clot lodged in the right side of arterial Circle of Willis including the middle and posterior cerebral arteries (arrows). This photograph was taken from a rat that died 12 h after embolization with 6 cm clot.

After recovery from the anesthesia for implantation of the indwelling catheter, each rat was examined carefully for the presence of neurological signs related to the surgical procedure. Sixty minutes were allowed for complete recovery. By then, MABP had returned to normal, and the rats had resumed their normal behavior such as grooming, eating, and pacing around inside the cage. The pre‐loaded single blood clot, with its proximal end positioned at the 7 cm marker, was slowly injected over 10 s into the ICA with 15 μL of normal saline using a 100 μL Hamilton syringe. An additional 10 μL of normal saline was injected to ensure a complete discharge into distal ICA. The catheter was occluded with a vascular clip and severed. An example of the clot localization is shown in Figure 1.

### 2.4 Study protocol

All rats were randomly assigned to three major groups, 30 rats per group, to receive a single blood clot of either 1.5, 3, or 6 cm in length according to the study protocol. Each major group was further divided into two subgroups, 15 each, for assessment of brain infarction and determination of water content. To minimize the variability caused by clot inequality, the experiment was carried out in a set of three, one for each of the three groups, using the same batch of blood clots. Each set had its own new batch of clots. For the brain water content control study, 11 rats that had all surgical procedures, except for clot injection, served as surgical sham controls. Normal brain water content was studied in another 11 rats, without any surgical intervention.

### 2.5 Mortality rate, neurological deficits, and time course of changes in MABP

Each rat was observed for 24 h after injection of the blood clot or until death. The general appearance and behavior were recorded at 1, 4, 8, 16, and 24 h after injection of the blood clot. The MABP was recorded every 5 min for the first hour, every 10 min for the second and third hour, every 30 min for the fourth to eighth hour and every 60 min thereafter until completion of the 24 h study or until death.

General appearance and neurological deficit scores (NDS) were recorded according to Tsai et al. (2014): 0, no neurologic deficit; 1, one paw clumsiness or clasping during tail suspension; 2, tilting; 3, rounding in a unilateral circle; 4, akinesia; 5, seizure; 6, stupor. A score was assigned for each or several possible spontaneous symptoms and, if several were noted, the scores were added.

### 2.6 Brain infarction

Survivors at 24 h following embolization were decapitated under deep isoflurane anesthesia, while non‐survivors were prepared at time of death. The brains were procured, examined, and sliced into seven 2‐mm thick coronal sections for 2,3,5‐triphenyltetrazolim chloride (TTC) staining and quantification via standard photography and digital image analysis. The infarcted area was numerically integrated across each section and over the entire ipsilateral hemisphere. The infarcted volume was measured separately in the cortex, caudate‐putamen, and hemisphere, and was expressed as volume percentage of the contralateral structure.

### 2.7 Brain water content

Brain water content in left (non‐ischemic) and right (ischemic) hemispheres was assessed 24 h following embolization, or at time of death if the rat died before 24 h. Eleven animals served as a surgical sham control group. These rats received all surgical procedures as the study groups, but without blood clot injection. Their brains were removed 24 h after surgery. Another 11 rats served as naive control for brain water content measurements. These rats were killed by decapitation under deep anesthesia. The brain was quickly removed and divided into right and left hemispheres. The brain was weighed immediately to obtain wet weight (WW) and then dried at 100°C for 24 h to obtain the dry weight (DW). The percentage of brain water content was calculated as 100 × (WW − DW)/WW.

### 2.8 Statistical analysis

Differences in survival were analyzed with the log‐rank Kaplan–Meier survival analysis. MABP was analyzed by two‐way repeated measures ANOVA where embolic clot size was a between‐subject factor and time was a within‐subject factor. Infarct volume and brain water content were analyzed by one‐way ANOVA. If the effect of embolic size was significant, the Holm–Sidak procedure for multiple comparisons was applied to compare individual groups. Because the NDS score consisted of discreet integers, the non‐parametric Kruskal–Wallis test was used for analysis of ranks among groups at each time point. When there was a significant group effect, individual groups were compared by the post hoc Dunn's test. In all cases, the significance level was set at *p* < 0.05. Data are presented as means ± SD or as medians with quartiles. Sigmaplot 12.5 software was used for statistical analysis and construction of graphs.

## 3 RESULTS

### 3.1 Mortality

**FIGURE 2:**
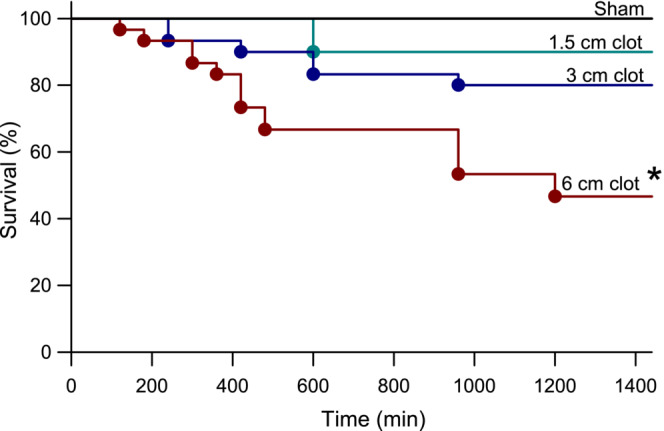
Time course over 24 h of percent survival in rats undergoing sham surgery (*n* = 12) or internal carotid artery injection of a blood clot 1.5, 3, or 6 in length (*n* = 30 per group). Survival rate in group receiving the 6 cm clot was significantly lower compared to the other groups by the Kaplan–Meier survival log‐rank test and Holm–Sidak procedure for multiple comparisons.

Survival to the 24‐h endpoint occurred in 12 of 12 sham‐operated rats, 27 of 30 rats embolized with a 1.5 cm clot (90%), 24 of 30 rats embolized with a 3 cm clot (80%), and 14 of 30 rats with a 6 cm clot (47%). The Kaplan–Meier survival log‐rank test indicated a significant effect among groups (*p* < 0.001), and the Holm–Sidak procedure indicated that survival in the 6 mm clot group differed significantly from the three other groups (Figure 2).

### 3.2 Neurologic deficit scores and neurologic behavior

**FIGURE 3:**
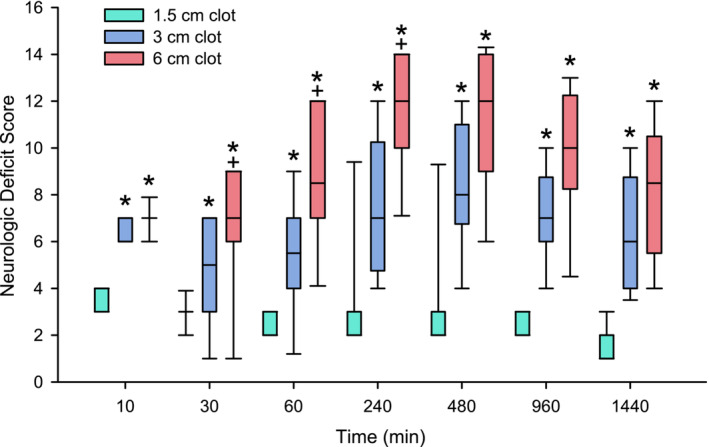
Neurologic deficit scores were graded according to clot size. Box plots of 5, 25, 50, 75, and 95 percentiles of neurological deficit scores assessed over 24 h after internal carotid artery injection of a blood clot 1.5, 3, or 6 cm in length. Because of early mortality, sample size decreased from 30 to 28 by 1440 min in the 1.5 cm clot group, from 30 to 24 by 1440 min in the 3 cm clot group, and from 30 to 14 by 1440 min in the 6 cm clot group. *p < 0.05 from 1.5 cm length clot; + *p* < 0.05 from 3 cm length clot by the Kruskal–Wallis test and Dunn's test for multiple comparisons.

The neurologic deficit scores following injection of the clot into ICA are presented in Figure 3. Deficit scores in the 3 and 6 cm clot groups were significantly worse than those in the 1.5 cm clot group from 10 min through 1440 min. Deficit scores in the 6 mm clot group were significantly worse than those in the 3 mm clot group from 30 min through 240 min. After 480 min, a small diminution of the scores was attributable to loss of the rats with the worse deficits. Overall, changes in the neurologic deficit scores varied with embolic size.

Although the neurologic deficits produced by embolization in rats were variable, the initial clinical presentation of their neurobehavior was generally similar. Immediately after clot injection, most of the rats became irritable, with erratic behavior for a few seconds followed by cessation of all motor activity for 15–20 min. Deviation of the head and neck toward the side of embolization generally developed within 1–2 h. Weakness of the limbs, contralateral to the side of embolization as evidenced by circling movement, developed at a later time of 2–3 h after embolization. Atypically, 5 of the 30 rats in the 6 cm clot group dramatically lost the upright posture soon after injection of clot, fell on their side, and became unconscious. These rats slowly emerged 20–40 min thereafter and were lethargic but with sustained upright position. All of these rats died between 8 and 16 h following clot injection. Later, some rats developed generalized seizures followed by death, while others recovered. Fifteen of thirty rats in the 6 mm clot group, 5 of 30 rats in the 3 mm clot group, and 2 of 30 rats in the 1.5 mm clot group developed severe seizures within 40–240 min after embolization. Seizure activity continued intermittently until their death. Only two rats (one each in the 1.5 and 3 mm clot groups) that had a late seizure onset (150 min or later) survived.

### 3.3 Time course of change in MABP

**FIGURE 4:**
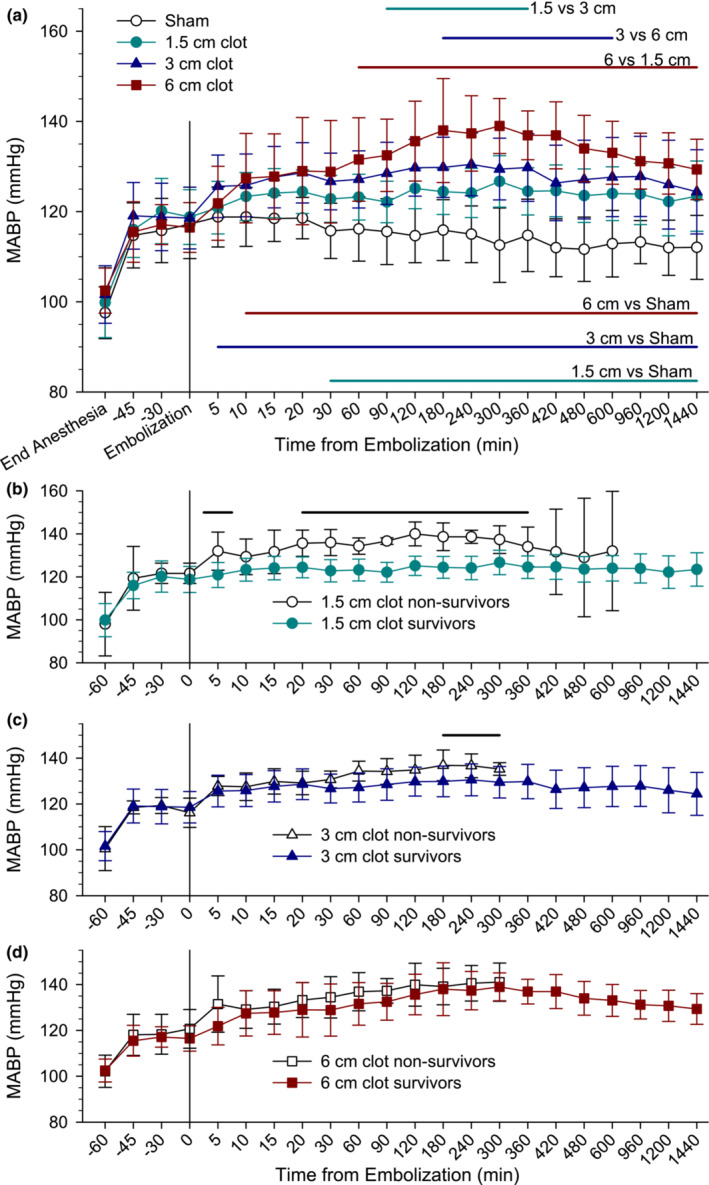
Increase in mean arterial blood pressure (MABP) was graded to the size of the embolus. (a) Time course of MABP over 24 h (1440 min) in rats that survived 24 h after a single clot embolization of 1.5, 3, and 6 cm in length and in rats undergoing sham surgery. Horizontal lines indicate time points at which group comparisons were significant (*p* < 0.05) by two‐way ANOVA with repeated measures and the Holm–Sidak procedure for group comparisons. Comparison of MABP in 24‐h survivors and those that died before 24 h after embolization with a 1.5 cm clot (b), 3 cm clot (c), and 6 cm clot (d). Data are presented as mean ± SD.

The time course of MABP is shown in Figure 4a for all rats that survived to the end‐point at 24 h. Two‐way repeated measures ANOVA indicated significant effects of group, time, and their interaction (*p* < 0.001). MABP increased approximately 15–20 mm Hg within 15 min of ceasing isoflurane administration and was equivalent in all groups at the time of embolization (60 min after ending anesthesia). In the sham‐operated rats, MABP remained stable over the subsequent 1440 min of monitoring, whereas clot injection produced early increases in MABP. Interestingly, further increases that were graded to the size of the clot were noted between 30 and 300 min. The increases became significantly different from the sham‐operated group by 30, 5, and 10 min after embolization in the 1.5, 3, and 6 cm clot groups, respectively, and these differences remained significant throughout the 1440 min of monitoring. Moreover, MABP in the 3 cm clot group was greater than that in the 1.5 cm clot at 90–360 min, MABP in the 6 cm clot group was greater than that in the 3 cm clot group at 180–600 min, and MABP in the 6 mm clot group was greater than that in the 1.5 cm clot group at 60–1440 min.

Because the non‐survivors had missing MABP values, they were not included in the overall repeated measures ANOVA; they were compared separately over the duration for which all had survived for the particular clot size. The non‐survivors had higher MABP than the survivors at 5 min and at 20–360 min in those embolized with a 1.5 cm clot (Figure 4b), and at 180–300 min in those embolized with a 3 cm clot (Figure 4c). In those embolized with a 6 cm clot, MABP did not differ between those that survived 24 h and those that did not (Figure 4d).

### 3.4 Brain infarction

**FIGURE 5:**
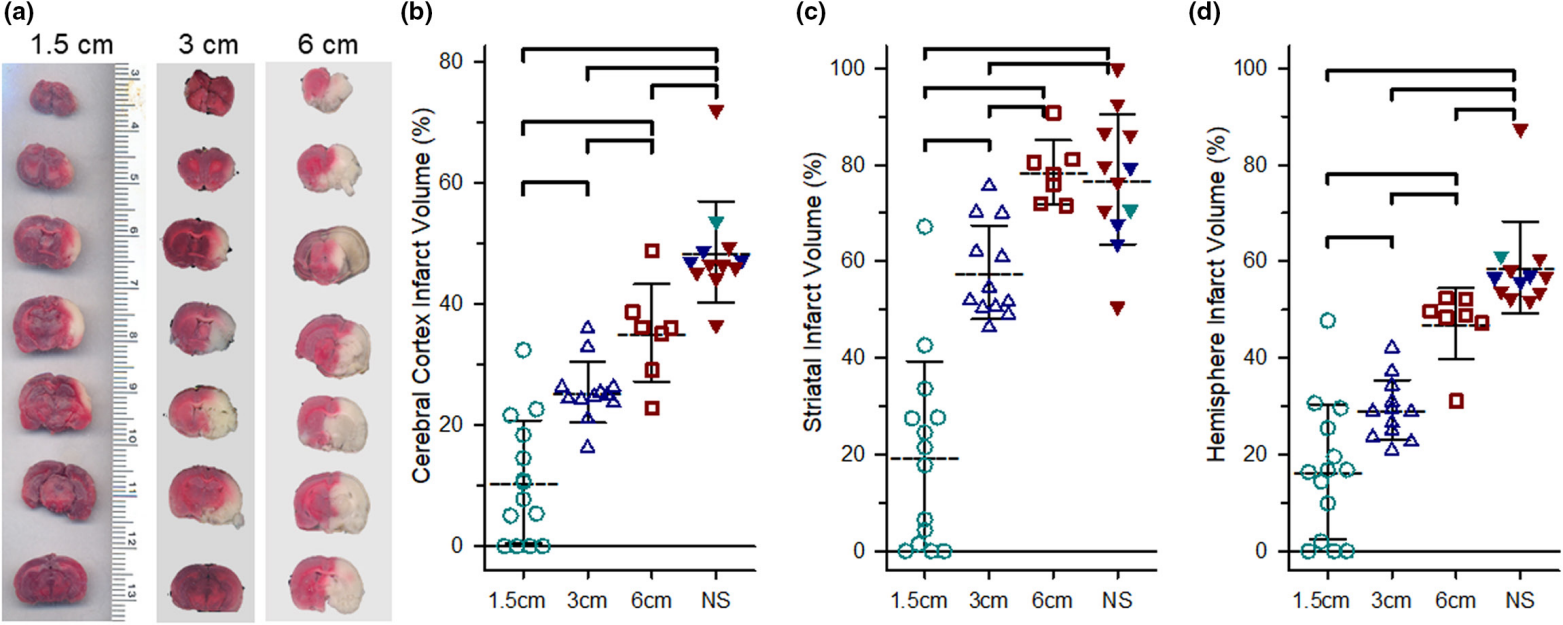
Infarct volume was graded to size of embolus in 24‐h survivors and greatest in non‐survivors. Representative images of coronal brain sections stained with tetrazolium chloride from rats embolized with 1.5, 3, and 6 cm long clots (a). Infarct volume in cortex (b), striatum (c), and entire hemisphere (d) in rats that survived 24 h. Those that did not survive (NS) but in which the brain was harvested soon after death were analyzed separately and data were pooled from the 1.5, 3, and 6 cm embolized groups. Lines indicate means ± SD. Brackets indicate paired comparisons that were significantly different (*p* < 0.05) by one‐way ANOVA and the Holm–Sidak procedure for multiple comparisons.

For sham operated animals, there was no visible infarct lesion, as in normal controls. Representative images of TTC‐stained brain sections are shown in Figure 5. In those that survived 24 h, increases in embolus size led to progressive increases in infarct size in cerebral cortex (10.6% ± 10.1%, 25.5% ± 5.0%, and 35.2% ± 8.0% of cortex with 1.5, 3, and 6 cm clot, respectively), in striatum (19.6% ± 19.7%, 57.7% ± 9.7%, and 78.5% ± 6.6% of striatum) and in the entire hemisphere (16.4% ± 13.9%, 29.2% ± 6.2%, 47.1% ± 7.3% of hemisphere; *n* = 14, 12, and 7 survivors for 1.5, 3, and 6 cm clot groups, respectively). One‐way ANOVA indicated a highly significant effect of clot size on infarct volume for each of the three regions (*p* < 0.001). Post hoc multiple comparisons with the Holm–Sidak procedure indicated that infarct volume in each of the groups differed significantly from the other two groups (Figure 5). In non‐survivors, brain injury was more pronounced, and no differences were observed in the non‐survivors among the 1.5 cm (*n* = 1), 3 cm (*n* = 3), and 6 cm (*n* = 8) clot groups in cerebral cortex (53.6%, 47.7% ± 0.9%, 48.2% ± 10.3% of cortex, respectively), in striatum (70.6%, 70.3% ± 8.2%, 80.3% ± 15.1% of striatum, respectively), and hemisphere (61.1%, 56.5% ± 0.7%, 59.3% ± 11.7% of hemisphere, respectively). Thus, for comparison with survivors, the non‐survivors from the three clot sizes were pooled into a single group for statistical analysis. As shown in Figure 5, the non‐survivors had significantly larger infarcts than the survivors from all three clot size groups in cerebral cortex and hemisphere. In striatum, infarcts in the non‐survivors were larger than those in the survivors from the 1.5 cm and 3 clot groups, but not from the survivors from the 6 cm clot group.

**FIGURE 6:**
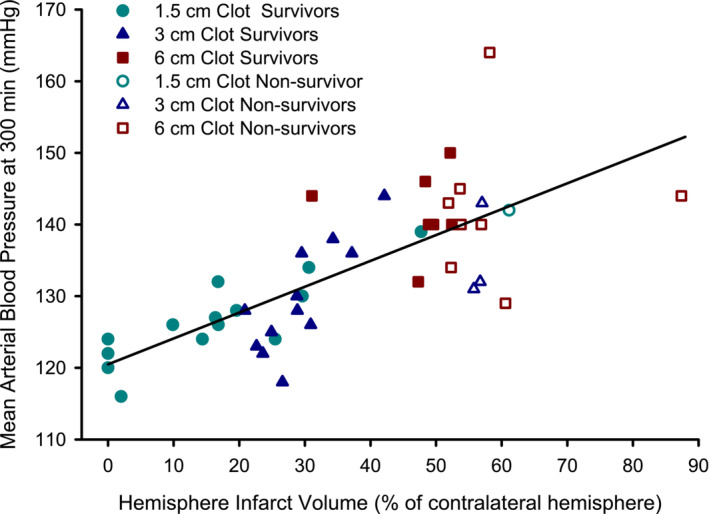
Pressor response correlates with infarct volume. Mean arterial pressure at 5 h after clot injection, when hypertension was greatest, is plotted against the hemisphere infarct volume measured at 24 h or at the time premature death for groups injected with 1.5, 3, and 6 cm clots. Regression line, shown for data pooled from all groups, had a correlation coefficient of 0.737 (*r* ^2^ = 0.54; *p* < 0.001).

Because both infarct volume and the pressor response after clot injection depended on clot size, we examined whether there was a relationship of the pressor response with infarct volume (Figure 6). We used the MABP values at 5 h because that was the approximate time when MABP reached a peak value. When pooling the data from all of the groups, MABP at 5 h correlated with the total hemisphere infarct volume (*r* ^2^ = 0.54; *p* < 0.001). This correlation suggests that the pressor response in the early hours after stroke is related to the volume of tissue undergoing cell death.

### 3.5 Brain water content

**FIGURE 7:**
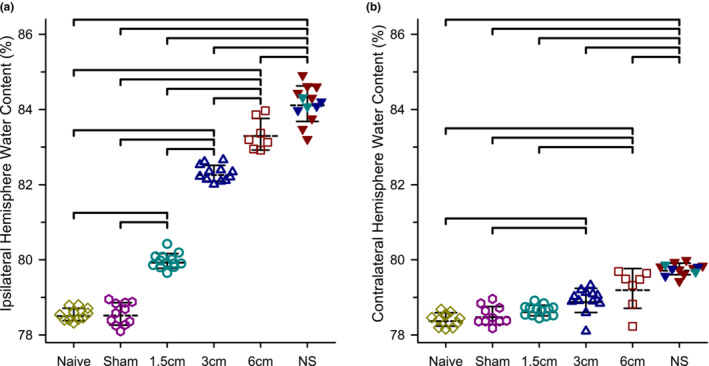
Increases in hemispheric brain water content was graded to the size of the embolus in 24‐h survivors and was greatest in non‐survivors. Results are shown for a group of naïve rats with no surgery, a group undergoing sham surgery, groups that survived embolization with a blood clot 1.5, 3, or 6 cm in length, and those that did not survive (NS) embolization with any size clot. Percent water content was calculated from wet and dry weights of right hemisphere (a, ipsilateral to embolization) and left hemisphere (b, contralateral to embolization). Lines indicate means ± SD. Brackets indicate paired comparisons that were significantly different (*p* < 0.05) by one‐way ANOVA and the Holm–Sidak procedure for multiple comparisons.

For sham operated animals, brain water content was similar to that in non‐operated naive group for the hemisphere ipsilateral to the neck surgery (78.6% ± 0.3% in sham, *n* = 11; 78.5% ± 0.1% in naive, *n* = 11). Graded increases in embolus size led to progressive increases in water content in the hemisphere ipsilateral to the stroke for those that survived 24 h (80.0% ± 0.2%, 82.3% ± 0.2%, 83.3% ± 0.4% water content; *n* = 13, 12, and 7 survivors for 1.5, 3, and 6 cm clot groups, respectively; *p* < 0.001 overall group effect by one‐way ANOVA). The ipsilateral water content in each clot size group significantly differed from each other and from the control and sham groups (*p* < 0.001 by Holm–Sidak procedure; Figure 7). In the non‐survivors, water content was elevated in all embolized groups (84.2% ± 0.2%, 84.1% ± 0.1%, 84.2% ± 0.63% *n* = 2, 3, and 8 non‐survivors for 1.5, 3, and 6 cm clot groups, respectively) and appeared independent of clot size. When the non‐survivors were pooled into a single group for statistical comparison to survivors, the water content in the non‐survivors was significantly higher than that in each of the individual survivor groups. Interestingly, in the contralateral hemisphere, small increases in water content were detected in the 3 and 6 mm clot survival groups and in the combined non‐survival group compared to the control and sham groups (78.4% ± 0.2%, 78.5% ± 0.2%, 78.7% ± 0.1%, 78.9% ± 0.3%, 79.2% ± 0.5%, and 79.8% ± 0.2% for the naïve, sham, 1.5, 3, 6 cm, and combined non‐survivor groups, respectively). However, these increases were much smaller than those in the corresponding ipsilateral hemisphere (Figure 7).

## 4 DISCUSSION

To mimic more closely to the pathogenesis of a clinical embolic stroke, we induced acute ischemia by instilling a blood clot into the ICA in conscious, non‐anesthetized rats. This model allowed us to observe the immediate neurological behavior from the onset, and evaluate the relationship between cerebral ischemia and systemic hemodynamic changes. The study demonstrated: (1) embolization of the ICA can induce stroke acutely in the un‐anesthetized, awake rat; (2) by using a larger diameter and length of clot, the ischemic infarct lesion produced is less variable for given size of clot; (3) there is a clear link between systemic hemodynamic hypertensive response and cerebral ischemia.

Although this paradigm represents another embolic model for a focal ischemia, there are three features distinctively different from the conventional embolic model: (a) we used a large diameter blood clot injected into the ICA, (b) the contralateral CCA need not be occluded, as is usually done to help stabilize the injected blood clot in the model of Zhang, Chopp, et al. (1997), and (c) the blood clot is injected while the rat is conscious and freely moving, eliminating any possible anesthetic effect. With regard to the clot size, the clot was prepared from a PE 90 with an ID of 0.86 mm, yielding a larger diameter blood clot than that from PE 50 with an ID of 0.58 mm used in the conventional MCAo model. We assessed the clot size by filtering the clot through a PE 20 catheter with an ID of 0.38 mm to standardize the clot diameter. The diameter of the clot is in agreement with the sphere diameter size of 0.3–0.4 mm that is the best to occlude the distal ICA obtained by Gerriets et al. (2003) from a macro‐sphere model for permanent MCAo. Spratt et al. (2006) also found an occluding thread tip diameter of 0.37 ± 0.03 mm to be optimal for yielding a reproducible stroke size in the rat. In an average size rat (300 g), the size of the proximal ICA is 0.56 mm, and distally it is reduced to 0.3 mm or less as it bifurcates into the MCA (0.24 mm) and anterior cerebral artery (ACA, 0.28 mm; Scremin, 1995). Using a large diameter clot permits a greater chance of lodging at the terminal ICA, thereby occluding the MCA. The embolic MCAo model developed by Zhang, Chopp, et al. (1997) used a clot preparation from PE‐50 that yielded a clot with a smaller diameter (PE10 with ID 0.28 mm). With this smaller size clot, a greater chance exists of the clot being washed away rather than lodging at the terminal ICA around the orifice of MCA. This possibility could account for the considerable variability of the ischemic infarct associated with this model, even when the contralateral CCA is occluded for 30 min to reduce pressure in the Circle of Willis.

In our early attempts to use the protocol of Zhang, Chopp, et al. (1997), with a clot diameter of <0.28 mm, infarction was inconsistent. An additional factor is that placement of the PE‐50 delivery catheter into the distal ICA near the proximal MCA requires stretching the tip so that the outside diameter is approximately 0.3 mm. One limitation of this process is the difficulty to consistently stretch the tubing without changing the circular shape of the lumen. With the availability of PE‐10 and PE‐8 tubing, stretching is not required. We subsequently tested PE‐10 tubing (OD 0.63 mm, ID 0.28 mm; Papangelou et al., 2013) and more recently to PE‐8 tubing (OD, 0.36 mm, ID, 0.2 mm; Chuang et al., 2018) for the clot delivery catheter. However, with the smaller diameter tubing, we had to increase the length of blood clot from 30 mm to 55 mm to obtain reasonably‐sized infarcts. Likewise, Asahi et al. (2003) found it necessary to increase the clot length to 50 mm when using a PE‐10 size catheter. The disadvantage of this refinement is that the smaller diameter of the clot can be smaller than the diameter of the artery, and therefore increases the likelihood that fragments of the clot could be carried more distally and produce more variable regions of ischemia. In the original model, blood flow from contralateral CCA has to be temporarily stopped (20–25 min) to help stabilize the position of the embolus (Beech et al., 2001; Zhang, Chopp, et al., 1997). We have overcome these limitations by delivering a larger diameter embolus through a PE‐20 catheter into the ICA to ultimately occlude the distal ICA while avoiding temporary occlusion of the contralateral CCA.

### 4.1 Comparison with other established MCAo models

**FIGURE 8:**
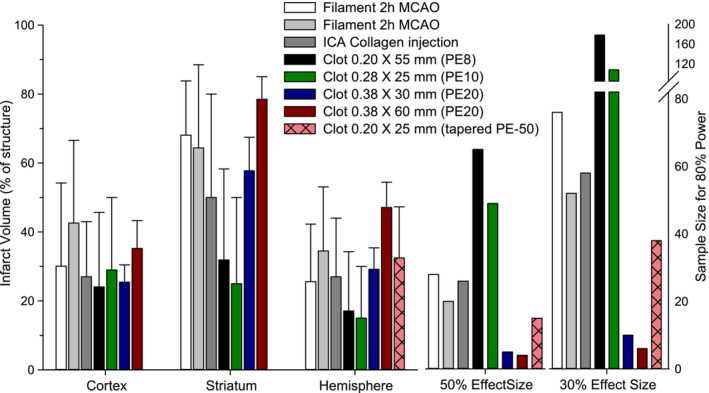
Comparison of mean and SD of infarct volume in cerebral cortex, striatum, and total hemisphere and estimated sample size needed to achieve 50% and 30% effect sizes in various rat models of ischemic stroke. Volumes of infarcted brain tissue were the largest utilizing the present model with a 60 mm embolus. By using a smaller clot, 30 mm in length, the extent of injury is similar with other models but the SD is small, resulting in smaller sample size requirements for 80% power.

Cerebral infarct lesion produced by the present model was compared with the published data in rat from our laboratory based on the well‐established filament model with 2 h of MCAo (Alkayed et al., 1998; Zhang et al., 2012), an atherothrombotic model involving injection of collagen into the ICA to activate platelets (Schunke et al., 2015), and embolic models in which the clot was injected into the ICA via a PE‐8 catheter (Chuang et al., 2018) or a PE‐10 catheter (Papangelou et al., 2013). Using the 30 mm length clot formed in PE‐20 tubing in the present study, the mean infarct volume in cerebral cortex, striatum, and the entire hemisphere was comparable to that obtained with the transient filament model performed by two different surgeons and by the collagen injection model (Figure 8). In contrast, embolization with the smaller diameter clots that utilized the PE‐8 and PE‐10 catheters resulted in a smaller mean infarct values with a greater SD. Interestingly, the mean hemisphere infarct volume with the 30 mm‐length clot was similar to that reported in the original rat embolic model by Zhang, Chopp, et al. (1997) with a 25 mm long clot injected through a tapered PE‐50 catheter with an estimated clot diameter of 0.2 mm. However, the SD in the current model is about half of that reported in the original model. This smaller variability might be attributed to our use of a larger diameter clot, the avoidance of anesthesia during MCAo onset, and the necessity of keeping the CCA permanently occluded to avoid re‐anesthetizing the rat after MCAo.

To evaluate the impact of a smaller SD on the ability to detect a cerebroprotective intervention, we performed a sample size calculation for detecting either a 50% or 30% change in infarct volume with 80% power (*α* = 0.05; 2‐tail). The effect size was calculated as a percentage of the mean value for each model rather than using the same absolute volume for each model because power analysis typically uses the mean value for that particular model. The sample sizes vary greatly across models, with the largest values in our previous embolic models with small diameter clots (Figure 8). The original model of Zhang, Chopp, et al. (1997) outperformed our earlier models, whereas the modifications made in the present model resulted in a small required sample size (*n* = 5 for a 50% effect size; *n* = 10 for a 30% effect size with a 30 mm length clot).

When the clot length was extended to 6 cm in the present study, the infarction extended over approximately half of the cerebral hemisphere and exceeded the infarct size seen in the other MCAo models yet the SD and power analysis remained favorable (Figure 8), likely because perfusion through the anterior and posterior cerebral arteries was also reduced. The infarction was associated with massive edema and a high mortality, thereby mimicking the malignant stroke observed clinically in ICA stroke syndromes (Hacke et al., 1996). Regardless of embolic length, all deceased animals also had the common pathophysiological findings of large hemispheric infarcts, massive increase in brain water content, and a sustained elevation of MABP. This suggests that these animals may have succumbed to severely increased intracranial pressure. Clinically, patients with severe intracranial hypertension caused by a massive stroke have been successfully treated with osmotherapy or surgical decompressive craniectomy (Kalia & Yonas, 1993; Suarez et al., 1998). Thus, this model with the 60 mm clot could be an ideal model to evaluate experimentally such therapeutic measures for malignant stroke.

It is noteworthy that many of the non‐survivors, especially those in the 60 mm clot size group, developed seizure activity that often was followed by death. Seizures are not commonly reported in the other ischemic stroke models. The occurrence of seizures in our model could be attributed to the presence of large infarctions in non‐survivors. In addition, the rats in our study were closely observed throughout the entire 24‐h survival period, whereas animals in many stroke studies are not observed during the night and behavior prior to early death is not always captured.

### 4.2 Blood pressure response

Systemic hypertension occurred in response to embolization. The time of blood pressure elevation and the magnitude of hypertension, however, varied with the size of embolus and the resulting cerebral ischemic changes. This hemodynamic response is presumed to be a response by the central nervous system to increase cerebral perfusion pressure to restore CBF, but this rise did not necessarily correlate with the outcome of ischemia. When the time course of changes in MABP in deceased animals were analyzed separately, as compared to survivors, it showed that blood pressure rose significantly following embolization, accelerating steadily over time until death. There were no statistical differences in the degree of hypertension in non‐survivors among groups. In comparison, MABP in survivors from all groups demonstrated similar patterns of change, that is, a significant but a smaller rise following embolization, a slower rate of increase over time, and a gradual decline after 5–6 h of embolization. Compared to the pre‐ischemic state, blood pressure nevertheless remained significantly elevated at 24 h after embolization. Our finding of MABP trends support the clinical observation that blood pressure is elevated in most ischemic stroke patients upon hospital admission but declines by the tenth day in the majority of survivors (Wallace & Levy, 1981), whereas continued marked elevation of blood pressure during the early phase of acute cerebral ischemia portends a worse ischemic outcome (Kalia & Yonas, 1993). Indeed, we found a significant correlation between the MABP at 5 h after stroke onset with infarct volume. This correlation suggests that the magnitude of the pressor response is proportional to the volume of tissue subjected to ischemia.

Injection of the clot produced an immediate brief period of irritable, erratic behavior that was usually followed by a quiet period of no activity lasting 15–20 min. Thus, the increase in MABP in this 15–20 min‐period was not attributable to excessive motor activity. Indeed, when rats regained spontaneous activity, they were generally less active than those in the sham group. Although we did not investigate the precise mechanisms of the pressor response, the rapid increase noted within 5–10 min of MCAo presumably is the result of increased sympathetic outflow. Activation of the renin‐angiotensin system and release of vasopressin might also contribute to the subsequent gradual rise in MABP over the ensuing 5 h.

Another consideration for the secondary, gradual rise in MABP is that the ischemic core likely expands over time as regions in the electrically silent penumbra begin to undergo depolarization from sustained inadequate oxygenation and waves of spreading depolarization. In global cerebral ischemia, the threshold for the pressor response corresponded to a 30%–40% reduction of cerebral blood flow at which point oxygen extraction is maximal and cerebral oxygen consumption began to fall; below this threshold, the magnitude of the pressor response correlated with the percent reduction of cerebral oxygen consumption (Koehler et al., 1989). In the case of focal ischemia, it is possible that the expansion of tissue with decreased oxygen consumption and with consequent anoxic depolarization leads to a greater pressor response over time. In previous work where anesthesia continued for 30–60 min after clot embolization, the pressor response was absent until anesthesia was discontinued (Chuang et al., 2018). Thus, anesthesia interferes with the ischemic pressor response that would act to increase collateral blood flow. In support of this premise, infusion of phenylephrine during MCAo in anesthetized rats was found to increase intraischemic cerebral blood flow and decrease acute infarct volume (Drummond et al., 1989).

### 4.3 Limitations of the model and the study

There are a few limitations to this model: (1) the blood clot is instilled into the ICA while the ipsilateral CCA blood flow is blocked. The injected clot depends on a calculated amount of saline to flush into the arterial catheter and to be carried by blood flow from posterior circulation to the distal ICA; (2) the ipsilateral CCA blood flow is permanently occluded. However, this aspect of the technique is similar to the “filament model” of MCAo, where the ipsilateral CCA is often left occluded. A potential disadvantage of performing the MCAo in the awake state with a large clot in our model is that we permanently ligated the ipsilateral CCA for placement of the indwelling catheter rather than use a catheter inserted through the ECA. Because we wanted to avoid re‐anesthetizing the rat, we did not remove the indwelling catheter and ligature around the CCA. It is possible that permanent CCA occlusion could produce areas of hypoperfusion beyond the regions destined to infarct.

In addition to these limitations of the model, our study was limited in that we did not measure cerebral blood flow, brain electrical activity, or the efficacy of tissue plasminogen activator in dissolving the clots of this size. Measurements of cerebral blood flow in rats with imaging techniques or laser‐Doppler require immobilization and anesthesia, which we intended to avoid with the new stroke technique. We did not measure body temperature and cannot exclude the possibility that the large strokes induced hyperthermia, which could have exacerbated the injury and possibly was a factor in promoting seizures. Another limitation is that we did not extend survival beyond 24 h to determine the persistence of neurological deficits or performance on complex neurobehavior tasks. Also, we restricted the study to males. It is possible that the pressor response would be less in young female rats and that the protective effect of estrogen or other sex‐dependent factors would result in smaller infarcts in this model, as has been reported in the filament model (Alkayed et al., 1998). Lastly, we did not directly test the effects of anesthesia on infarct volume and edema with the exact same clot dimensions used in the current study. However, previous work with a smaller diameter clot showed that extending the duration of anesthesia after embolization worsened mortality and cerebral edema (Chuang et al., 2018). It is likely that anesthesia would also adversely affect mortality and edema formation with the currently used large diameter clots.

In summary, a new model is described utilizing a large diameter blood clot instilled into the ICA in a conscious, freely moving rat, to mimic the pathogenesis of a clinical embolic stroke. Use of a 3 cm‐long clot resulted in an infarction comparable in size to that obtained with 2 h of MCAo in the filament model but with a smaller SD, thereby providing good statistical power after allowing for a 20% mortality at 1 day. Use of a 6 cm‐long clot, produces a large cerebral infarction, severe edema, and a high mortality. This model of “malignant” stroke could be used as an ideal model for the study of medical therapeutic measures such as osmotherapy that promote reduction in the associated edema and intracranial mass effects as a consequence of grave cerebral ischemia. There is a clear link between systemic arterial blood pressure responses and the extent of cerebral ischemia. Continued elevation of blood pressure during the acute stage of ischemia appears indicative of a poor outcome. Furthermore, while this model has been designed to mimic embolic stroke, others have injected thrombin and collagen into the ICA of anesthetized animals to model thrombotic stroke (Chen et al., 2015; Schunke et al., 2015; Zhang, Zhang, et al., 1997). The surgical procedures used herein for producing embolic stroke could also be adapted for producing thrombotic stroke in awake rats and thereby cover a broader range of stroke etiology.

## AUTHOR CONTRIBUTIONS

Thomas J. K. Toung, Marek Mirski, and Raymond C. Koehler conceptualized the study. Thomas J. K. Toung refined the experimental protocol, performed the experiments, and accrued the data. NM assisted with the data accrual. Raymond C. Koehler performed the formal data analysis and created the graphic visualization of the data. Thomas J. K. Toung and Raymond C. Koehler wrote the manuscript and Marek Mirski helped edit the manuscript.

## FUNDING INFORMATION

This work was supported by National Institutes of Health grants R01 NS038684, R01 NS060703, and R01 HL139543 (to RCK).

## CONFLICT OF INTEREST STATEMENT

The authors declare that they have no financial or non‐financial interests to disclose and no conflict of interest.

## ETHICAL APPROVAL AND CONSENT TO PARTICIPATE

Not Applicable.

## HUMAN AND ANIMAL ETHICS

All experiments performed on animals were approved by the Animal Care and Use Committee of the Johns Hopkins University and were performed in accordance with the Guide for the Care and Use of Laboratory Animals (U.S. Department of Health and Human Services).

## CONSENT FOR PUBLICATION

All authors have read and approved the publication of this manuscript.
